# Nonpharmacological home remedies for upper respiratory tract infections: a cross-sectional study of primary care patients in Switzerland and France

**DOI:** 10.1093/fampra/cmad084

**Published:** 2023-08-13

**Authors:** Paul Sebo, Neria E Winkler, Mohamed Amir Moussa, Dagmar M Haller, Hubert Maisonneuve

**Affiliations:** University Institute for Primary Care (IuMFE), University of Geneva, Geneva, Switzerland; University Institute for Primary Care (IuMFE), University of Geneva, Geneva, Switzerland; Department of Cardiology, University Heart Center Zurich, University Hospital Zurich, Zurich, Switzerland; University Institute for Primary Care (IuMFE), University of Geneva, Geneva, Switzerland; University Institute for Primary Care (IuMFE), University of Geneva, Geneva, Switzerland; University Institute for Primary Care (IuMFE), University of Geneva, Geneva, Switzerland

**Keywords:** common cold, cough, home remedy, primary care, sore throat, upper respiratory tract infection

## Abstract

**Background:**

Many patients might be tempted to use nonpharmacological home remedies (NPHRs) to relieve upper respiratory tract infection (URTI) symptoms. However, primary care physicians (PCPs) rarely recommend NPHRs due to a lack of knowledge in this field. We conducted a questionnaire-based survey among primary care patients in Switzerland and France to explore which NPHRs they use and consider effective for 3 common URTI symptoms: sore throat/cough/common cold.

**Methods:**

Using official physician registries, we randomly selected 50 PCPs in Geneva (Switzerland) and Lyon/Grenoble (France). Seven research assistants were involved in the recruitment of consecutive patients from the waiting rooms of these PCPs (20–25 patients per practice). Patients were asked to complete a paper-based questionnaire to assess the use and perceived effectiveness of 72 NPHRs for URTI symptoms. The list of NPHRs was developed by our research team with the help of 97 patients. Remedies were considered effective if patients reported that they were effective/very effective. Data were analysed descriptively.

**Results:**

Of the 1,198 eligible patients, 1,012 agreed to participate (84.5%). The 4 most frequently used NPHRs were honey/lemon/thyme/herbal teas. Most patients using these NPHRs considered them as effective (between 77% of patients for onion syrup for cough and 94% of patients for thyme inhalations for common colds).

**Conclusions:**

Many patients reported using honey/lemon/thyme/herbal teas for URTI symptoms, and generally considered these treatments to be effective. Future research should explore the extent to which these remedies can be safely proposed as alternatives for the symptomatic treatment of ear/nose/throat complaints in primary care.

**Key messages**

- The participation rate in this questionnaire survey of primary care patients was 85%.
- The most frequently used home remedies were honey, lemon, thyme, and herbal teas.
- Between 77% and 94% of patients using home remedies considered them to be effective.

## Introduction

Upper respiratory tract infections (URTIs) are a frequent reason for consultation in primary care.^1^ The vast majority of URTIs are viral in origin and are treated symptomatically, primarily with intranasal or oral decongestants, oral analgesics, and oral nonsteroidal anti-inflammatory drugs.^2^ Although the level of evidence for these drugs is low, they are claimed to reduce the intensity and/or duration of symptoms.^3^

There is little data on the effectiveness and side effects of nonpharmacological home remedies (NPHRs) for URTIs. There is some evidence that honey and thyme may be effective in treating URTI symptoms due to their antimicrobial and/or anti-inflammatory activity.^4,5^ In terms of side effects, the data are reassuring for honey, lemon, thyme, and herbal teas, with few side effects reported in adults.^6^ For inhalations, the classic method using a bowl of hot water and a towel could occasionally result in burns, especially in children.^7^

We recently showed that many patients might be tempted to use NPHRs instead of or in addition to pharmacological medications to relieve various ailments, such as pain, sleep disturbance, or constipation.^8^ However, to the best of our knowledge, only a limited number of guidelines currently advise to use NPHRs in primary care.^9–11^ In this context, it makes sense to observe that primary care physicians (PCPs) rarely recommend NPHRs, mainly due to a lack of knowledge in this field.^12^ As with any medical consultation, it can have a negative impact on the care process and the patient-physician relationship if PCPs do not explore and address their patients’ expectations.

We conducted a survey among primary care patients in France and Switzerland to explore which NPHRs they use and consider effective for 3 common symptoms of URTI (sore throat, cough, and common cold).

## Methods

### Setting and recruitment

This questionnaire-based survey was conducted between March 2020 and July 2021 in Switzerland (canton of Geneva) and France [6 departments grouped into 2 study sites: Lyon (departments of Rhône/Ardèche/Loire) and Grenoble (departments of Isère/Savoie/Haute-Savoie)]. It was part of a wider project conducted within our research group on the use of NPHRs by primary care patients and practitioners.^8,12–14^

We used the professional registers of community-based PCPs from the 3 regions (Geneva/Lyon/Grenoble) to form our sample frame from which we drew 50 PCPs at random, using simple randomization with computer-generated random numbers. These physicians were contacted by telephone or e-mail. If they refused or did not respond after 3 attempts, another physician from the list was drawn, and so on until 50 PCPs agreed to participate. In total, 168 PCPs were contacted (participation rate: 50/168 = 29.8%).

Seven research assistants were involved in the recruitment of consecutive patients from the waiting rooms of the selected PCPs (20–25 patients per practice). Patients under 18 years of age, those who did not understand French and those consulting for an urgent condition were excluded. The study was approved by the Research Ethics Committee of Geneva (Project-ID = 2020-00939).

### Development of the questionnaire and data collection

Patients were asked to complete a self-administered paper questionnaire in the waiting room to assess the use (Y/N) and perceived effectiveness (5 possible answers: ineffective, not very effective, effective, very effective, and do not know) of a large number of NPHRs (*n* = 304) for various medical conditions (*n* = 79).

The list of NPHRs was developed in 2 stages. First, we used a purposive sample of community-based PCPs in Geneva. We recruited consecutive patients from each practice and asked them to complete a questionnaire exploring which NPHRs they use and for which ailments. Patient recruitment was planned until saturation was reached (i.e. when 3 consecutive patients did not suggest any new remedies). A new practice was then tested, and so on until no new NPHRs were proposed. A total of 97 patients from 11 medical practices participated in the development of the prelist of 449 NPHRs. This prelist was then refined through discussions within our research team to obtain the final list used in the study.

The questionnaire and list of NPHRs for each medical condition are available as Supplementary Materials (French version: https://osf.io/5JK49/; English version: https://osf.io/nzt2q/). In the current study, we limited the analysis to 3 cardinal symptoms of URTIs: sore throat (*n* = 19 NPHRs), cough (*n* = 16), and common cold (*n* = 37).

There is no consensus definition of NPHRs. We defined NPHRs as remedies that cannot be purchased as commercially available drugs and that do not require external assistance from therapists.^8,12^

### Sample size calculation and data analysis

We calculated the sample size for an expected average prevalence of NPHR use of 60% with a 95% confidence interval (CI) of ± 5%. We included clustering in the calculation (intraclass correlation coefficient = 0.05, 20 patients recruited on average per PCP). The minimum sample size required was 720, but we decided to recruit 1,000 patients to account for missing data.

We summarized the socio-demographic data with proportions for categorical variables and medians and interquartile ranges for continuous variables. We used proportions and 95% CI adjusted for intracluster correlations within medical practices to summarize the data regarding use and perceived effectiveness of NPHRs. We used the ‘vce(cluster)’ option in Stata to obtain cluster-correlated robust variance estimates. Remedies were defined as ‘considered effective by patients’ if patients reported that they were either effective or very effective.

We calculated for each patient the proportion of NPHRs used that he/she considered effective/very effective. We assessed whether this proportion was associated with patient characteristics, using a generalized linear model with the logit link and the binomial family (univariable analysis: model adjusted for intracluster correlations; multivariable analysis: model adjusted for intracluster correlations and patient characteristics).

The statistical significance was set at a 2-sided *P*-value of ≤0.05. All analyses were performed with STATA 15.1 (College Station, TX).

## Results

Of the 1,198 eligible patients, 1,012 agreed to participate (84.5%), 61% of whom were women. Table 1 shows the participants’ main characteristics. Thirty-six percent of patients were recruited in Grenoble, 34% in Lyon, and 30% in Geneva. Their median age was 52 years. Eighty-four percent of patients reported being in good or excellent health.

**Table 1.** Patients’ characteristics (*N* = 1,012 patients).

Characteristic	*N* (%)	Median [interquartile range]
Female gender (*N* = 1,008)	616 (61.1)	
Age (*N* = 1,006)		52 [31]
Region (*N* = 1,012)		
Grenoble (France)	360 (35.6)	
Lyon (France)	345 (34.1)	
Geneva (Switzerland)	307 (30.3)	
Location of the PCP’s medical practice (*N* = 1,012)		
Urban zone	602 (59.5)	
Rural zone	410 (40.5)	
Nationality (*N* = 1,009)		
French	690 (68.4)	
Swiss	219 (21.7)	
Other	100 (9.9)	
Marital status (*N* = 1,004)		
Married or living as a couple	588 (58.6)	
Single	217 (21.6)	
Divorced or separated	127 (12.6)	
Widowed	72 (7.2)	
Work status (*N* = 1,009)		
Occupational activity	493 (48.9)	
Retired	324 (32.1)	
Student or apprenticeship/ vocational training	61 (6.1)	
Recipient of unemployment (ALV) or invalidity (DI) benefits ^1^	55 (5.5)	
Other	76 (7.4)	
Completed training (*N* = 1,009)		
University, FIT, UAS ^2^	354 (35.1)	
Intermediate school ^3^	479 (47.5)	
Compulsory schooling or no training/education	176 (17.4)	
Self-estimated general health status (*N* = 1,007)		
Excellent or very good	319 (31.7)	
Good	524 (52.0)	
Moderate or poor	164 (16.3)	
Number of daily medications (*N* = 995)		1 [13]
Number of visits to the PCP in the past 12 months (*N* = 1,010)		2 [0]

^1^ALV = unemployment insurance; DI = disability insurance.

^2^FIT = Federal Institute of Technology; UAS = University of Applied Sciences.

^3^Apprenticeship, vocational training, baccalaureate or diploma from intermediate school.

PCP, primary care physician.

Table 2 shows the 10 NPHRs most frequently used by patients for sore throat, cough and common cold, and their perceived effectiveness. The most frequently used NPHRs were honey, lemon, thyme, and herbal teas. About a quarter of patients used honey to treat all 3 symptoms of URTI. Different routes of administration were used, mainly gargles and teas for sore throats, teas for coughs, and inhalations and teas for common colds.

**Table 2.** The 10 NPHRs that are most frequently used by primary care patients for 3 common symptoms of URTI, and their perceived effectiveness (*N* = 1,012 patients).

Symptom and NPHR	Number of patients using the NPHR	Proportion of patients using the NPHR (95% CI)^1^	Number of patients who consider the NPHR effective or very effective among those who use it	Proportion of patients who consider the NPHR effective or very effective among those who use it (95% CI^1^)
Sore throat				
Honey gargles	264	26.1 (23.0–29.4)	242	91.7 (86.1–95.1)
Lemon gargles	160	15.8 (13.6–18.3)	139	86.9 (80.7–91.3)
Thyme infusions	139	13.7 (11.3–16.7)	124	89.2 (81.2–94.1)
Herbal teas	135	13.3 (11.2–15.8)	122	90.4 (83.4–94.6)
Tea gargles	77	7.6 (5.8–10.0)	68	88.3 (79.4–93.7)
Grogs	66	6.5 (5.1–8.3)	56	84.9 (72.7–92.2)
Milk	55	5.4 (4.2–7.1)	47	85.5 (72.8–92.8)
Silk scarf around the neck	55	5.4 (4.1–7.2)	47	85.5 (72.7–92.8)
Ginger	50	4.9 (3.6–6.8)	40	80.0 (66.0–89.2)
Curcuma	30	3.0 (2.1–4.1)	24	80.0 (62.0–90.8)
Cough				
Honey	249	24.6 (21.4–28.1)	217	87.2 (82.4–90.8)
Thyme infusions	120	11.9 (9.6–14.5)	111	92.5 (84.6–96.5)
Hot milk	78	7.7 (6.2–9.6)	67	85.9 (74.6–92.7)
Tea	72	7.1 (5.4–9.3)	65	90.3 (80.3–95.5)
Lemon	70	6.9 (5.4–8.8)	59	84.3 (72.0–91.8)
Ginger infusions	35	3.5 (2.3–5.2)	31	88.6 (69.8–96.3)
Cut onion on the bedside table	26	2.6 (1.7–3.9)	22	84.6 (57.5–95.7)
Sage infusions	25	2.5 (1.4–4.2)	21	84.0 (60.3–94.8)
Onion syrup	17	1.7 (1.0–2.8)	13	76.5 (49.8–91.4)
Garlic	14	1.4 (0.8–2.3)	11	78.6 (37.2–95.8)
Common cold				
Honey	260	25.7 (22.4–29.3)	231	88.9 (82.4–93.1)
Lemon	173	17.1 (14.7–19.8)	152	87.9 (79.9–93.0)
Thyme infusions	154	15.2 (13.1–17.6)	134	87.0 (79.2–92.2)
Herbal teas	139	13.7 (11.1–16.9)	122	87.8 (80.0–92.8)
Eucalyptus leaf inhalations	82	8.1 (6.5–10.1)	75	91.5 (83.2–95.9)
Grogs	81	8.0 (6.3–10.2)	66	81.5 (69.6–89.4)
Tea	79	7.8 (5.8–10.5)	66	83.5 (74.5–89.9)
Ginger	73	7.2 (5.2–9.9)	62	84.9 (74.0–91.8)
Hot milk	69	6.8 (5.3–8.7)	63	91.3 (81.8–96.1)
Thyme inhalations	63	6.2 (4.6–8.3)	59	93.7 (85.2–97.4)

^1^The 95% CIs were adjusted for intracluster correlations within medical practices.

For the majority of patients using them, all the NPHRs presented in Table 2 were perceived as effective (between 77% of patients for onion syrup for cough and 94% of patients for thyme inhalations for common colds). The lower bound of the 95% CI was ≥50% except for garlic for cough. There were no associations between the proportion of NPHRs considered effective/very effective and patient characteristics (Supplementary Appendix#1).

## Discussion

In summary, many primary care patients reported using honey, lemon, thyme, and herbal teas to relieve cardinal symptoms of URTI (sore throat, cough, or common cold) and generally considered these treatments to be effective.

Our findings are consistent with several studies conducted both in Europe and elsewhere.^15,16^ How these treatments work is not yet well understood. It appears that honey and thyme may be effective due to their antimicrobial and/or anti-inflammatory activity.^4,5^

There is little data on the risks of NPHRs. Although generally considered safe, NPHRs can rarely cause serious side effects. For example, burns directly related to steam inhalation have been reported, especially in children.^7^ If steam inhalations are to be used, with children it is recommended to advise parents to ‘sit in the bathroom with a hot shower running, the child on their lap, being read a story’.^17^

Our results demonstrate that NPHRs can offer an interesting alternative to pharmacological drugs for the relief of URTI symptoms and PCPs are therefore encouraged to explore their patients’ expectations in this area. Future research should assess the extent to which these remedies can be safely proposed as alternatives for the symptomatic treatment of ear, nose, and throat complaints in primary care.

### Limitations

The sample size was large and the study was carried out in 3 French-speaking European regions. However, the results of the study are not necessarily generalizable to other countries in Europe or elsewhere, as the NPHRs used may vary greatly according to region and cultural context. In addition, as in any questionnaire-based observational study, a certain degree of information bias is expected. There is a risk of bias especially for the perceived effectiveness of NPHRs, as we asked this question only to patients who had already used these remedies. However, it was not only frequent users who were questioned, but also those who rarely used them, perhaps because they considered them not effective enough.

## Conclusion

Many patients in the study reported using honey, lemon, thyme, and herbal teas to relieve URTI symptoms and generally considered them effective. Future research should explore the extent to which these remedies can be safely proposed as alternatives for the symptomatic treatment of ear, nose, and throat complaints in primary care.

## Supplementary Material

cmad084_suppl_Supplementary_Appendix

cmad084_suppl_Supplementary_Material

## Data Availability

The data underlying this article will be shared on reasonable request to the corresponding author.
