# Supplementary material for: Nonpharmacological home remedies for upper respiratory tract infections: a cross-sectional study of primary care patients in Switzerland and France
Source: Fam Pract. 2023 Aug 13;40(4):564–8. doi: 10.1093/fampra/cmad084 (PMC10667067; doi:10.1093/fampra/cmad084)
Supplement: cmad084_suppl_Supplementary_Appendix [file cmad084_suppl_supplementary_appendix.pdf]

Appendix#1. Associations between the proportion of non-pharmacological home remedies (NPHRs) considered effective or very effective for URTI symptoms, and patient characteristics (N=599 patients using at least one NPHR for URTI symptoms)

Characteristic	Unadjusted OR (95%CI)[1]	p-value	Adjusted OR (95%CI)[2]	p-value
Gender		0.81		0.91
Female	1.1 (0.7-1.6)		1.0 (0.7-1.5)	
Male	1		1	
Age [years]		0.07		0.07
< 40	1.7 (1.1-2.8)		1.8 (1.1-3.2)	
40-59	1.3 (0.8-2.0)		1.3 (0.8-2.1)	
≥ 60	1		1	
Location of the medical practice		0.06		0.07
Urban zone	1		1	
Rural zone	1.6 (1.0-2.5)		1.6 (1.0-2.6)	
Nationality		0.19		0.42
Swiss	1		1	
Other	1.4 (0.8-2.5)		1.3 (0.7-2.1)	
Completed training		0.37		0.23
University, FIT, UAS [2]	1		1	
Other	1.2 (0.8-1.7)		1.3 (0.9-1.9)	
Self-estimated general health status		0.69		0.90
Excellent or very good	1.4 (0.7-2.8)		1.2 (0.6-2.4)	
Good	1.2 (0.6-2.1)		1.0 (0.6-1.9)	
Moderate or poor	1		1	

[1]generalized linear model with logit link and binomial family (model adjusted for intra-cluster correlations within medical practices)
[2]generalized linear model with logit link and binomial family (model adjusted for intra-cluster correlations within medical practices and for all variables listed in the table)
[3]FIT = Federal Institute of Technology; UAS = University of Applied Sciences
